# Temporal and spatial variability of immunosuppressive therapies in transplant patients: An observational study in Italy

**DOI:** 10.3389/frtra.2022.1060621

**Published:** 2023-01-16

**Authors:** Maria Lucia Marino, Alessandro C. Rosa, Marco Finocchietti, Arianna Bellini, Francesca R. Poggi, Marco Massari, Stefania Spila Alegiani, Lucia Masiero, Andrea Ricci, Gaia Bedeschi, Francesca Puoti, Massimo Cardillo, Silvia Pierobon, Maurizio Nordio, Eliana Ferroni, Martina Zanforlini, Giuseppe Piccolo, Olivia Leoni, Stefano Ledda, Paolo Carta, Donatella Garau, Ersilia Lucenteforte, Marina Davoli, Antonio Addis, Valeria Belleudi

**Affiliations:** ^1^ Department of Epidemiology, Lazio Regional Health Service, Rome, Italy; ^2^ National Center for Drug Research and Evaluation, Istituto Superiore Di Sanità, Rome, Italy; ^3^ Italian National Transplant Center, Istituto Superiore di Sanità, Rome, Italy; ^4^ Azienda Zero of the Veneto Region, Padua, Italy; ^5^ Azienda Regionale per l'Innovazione e gli Acquisti, ARIA, S.p.A., Milan, Italy; ^6^ Regional Transplant Coordination, Milan, Italy; ^7^ Directorate General for Health, Milan, Italy; ^8^ General Directorate for Health, Cagliari, Italy; ^9^ Department of Clinical and Experimental Medicine, University of Pisa, Pisa, Italy

**Keywords:** immunosuppressive therapy, solid organ transplant, space–time variability, drug utilization, real-world evidence

## Abstract

**Background:**

In immunosuppression after transplantation, several multi-drug approaches are used, involving calcineurin inhibitors (*CNI: tacrolimus-TAC or cyclosporine-CsA*), antimetabolites (*antiMs*), mammalian target of rapamycin inhibitors (*mTORis*), and corticosteroids. However, data on immunosuppressive therapy by organ and its space–time variability are lacking.

**Methods:**

An Italian multicentre observational cohort study was conducted using health information systems. Patients with incident transplant during 2009–2019 and resident in four regions (Veneto, Lombardy, Lazio, and Sardinia) were enrolled. The post-transplant immunosuppressive regimen was evaluated by organ, region, and year.

**Results:**

The most dispensed regimen was triple-drug therapy for the kidneys [tacrolimus (TAC) + antiM + corticosteroids = 41.5%] and heart [cyclosporin  + antiM + corticosteroids = 36.6%] and double-drug therapy for liver recipients (TAC + corticosteroids = 35.4%). Several differences between regions and years emerged with regard to agents and the number of drugs used.

**Conclusion:**

A high heterogeneity in immunosuppressive therapy post-transplant was found. Further studies are needed in order to investigate the reasons for this variability and to evaluate the risk–benefit profile of treatment schemes adopted in clinical practice.

## Introduction

Immunosuppressive therapy is essential for transplant patients to ensure graft survival and to prevent rejection, with the aim of achieving a balance between graft tolerance and host defences. In general, the immunosuppressive protocol consists of two phases: induction therapy, administered to patients before or at the time of transplantation with the purpose of inducing severe and immediate immunosuppression, and maintenance therapy, a life-long therapy administered after transplantation (1).

During the maintenance phase, different drugs are usually administered, such as calcineurin inhibitors (*CNIs*), antimetabolites (*antiMs*), mammalian target of rapamycin inhibitors (*mTORis*), and corticosteroids (*Pred*).

From the pharmacological point of view, these drugs demonstrate the following mechanisms of action: CNI, cyclosporin (CsA), and tacrolimus (TAC) inhibit the production and release of interleukin-2 from activated T-helper cells by, in turn, inhibiting T-lymphocyte function (2), and they were the first immunosuppressors to be used in clinical practice; antiM, mycophenolate mofetil (MMF), and azathioprine (AZA) inhibit purine base synthesis required for B- and T-cell proliferation and represent the most common association with CNI; mTORis, sirolimus (SIR), and everolimus (EVE), the most recent immunosuppressors, work by inhibiting mTORis and interleukin2-driven T-cell proliferation, and they are used in combination with CNI and as an alternative for antiM; Pred, prednisone, methylprednisolone, and prednisolone block T-cell-derived and antigen-presenting cell-derived cytokine expression, and, together with CNI, they represent one of the mainstays of immunosuppressive therapy, although the long-term use of a high dose of steroids should be avoided in order to prevent potential side effects (3).

Therapeutic choice can vary according to organ setting, but usually it includes a combination of two or three drugs with the aim of reducing the doses of each drug and consequently the risk of adverse effects. The current standard immunosuppressive therapy in most kidney transplant protocols recommends a maintenance phase with CNI in combination with antiM or mTORi, with or without corticosteroids (4, 5). Kidney transplant represents the largest number of overall transplants and provides the strongest evidence on maintenance immunosuppressive therapies used in clinical practice. For other settings such as the liver and heart, evidence on the optimal immunosuppressive strategy is weak, despite therapeutic protocols being comparable with those adopted in kidney transplantation. In particular, in the liver setting, immunosuppressive regimens include a combination of CNI, antiM, or mTORi with or without corticosteroids (6, 7); in the heart setting, the standard immunosuppressive regimen is a combination of CNI, MMF, and corticosteroids, although to this triple therapy, EVE can be added in order to reduce the dose of CNI (8).

In recent decades, several comparisons between agents of the same therapeutic category have been made in various organ transplants. TAC-based therapy has emerged as a more effective therapeutic option than CsA, demonstrating better graft function, fewer rejections, and improved survival rates in kidney (9, 10) and liver (7, 11) transplants. In kidney transplantation, a large number of trials have shown fewer rejections of MMF than of AZA, co-administered with either CsA (12) or TAC (13); and the regimens comprising TAC and MMF with or without prednisone have become the “gold standard” in the management of this organ. MMF has thus supplanted the use of AZA by becoming the primary adjunctive agent in kidney transplant recipients (14). The introduction of mTORis on the market is relatively recent, and in Italy, it is currently approved, in combination with CsA or TAC, for kidney, heart, and liver transplant patients. Due to the synergistic immunosuppressive effects of mTORis and CNI, these combinations permit a reduction in the dose of CNI (15).

This evidence is summarized, for kidney transplantation, in the KDIGO clinical practice guidelines (4), which recommend a combination of therapies including CNI and antiM with or without Pred. In particular, KDIGO guidelines suggest TAC as a first-line CNI and MMF as an antiM. Moreover, the use of mTORis is suggested only when graft function is established, as a second-line therapy, or as an alternative option to standard regimens based on MMF + CNI (4, 15). The possible addition of Pred depends on the immunological risk of the recipient (4).

For the other organs, the guidelines are less specific for want of solid evidence. For liver transplantation, the combination of TAC and MMF exists and is often used in initial immunosuppressor regimens (7), but drug therapies including combinations of CNI, antiM, mTORi, and/or Pred are possible.

It is important to remember that the combination of different drugs and/or dosage can be adjusted to ensure suitable immunosuppression and to prevent adverse effects. Immunosuppressive regimens may vary across transplant centres and they should be tailored to patients’ needs. In particular, in the context of less frequent solid organ transplants such as the heart and the lungs, the clinical centre is the principal actor in the choice of therapies and its therapeutic protocols should take into account the patient's clinical history and more recent available evidence.

In the USA, the scientific registry of transplant recipients reports that triple therapy (TAC + MMF + Pred) is commonly used in all settings (16). In Asia, immunosuppressive regimens vary markedly across centres and organ settings (17). In Europe, and particularly in Italy, data on immunosuppressive therapy in clinical practice are scarce and, when available, mainly concern kidney transplant. The RECORD study reports that in south-eastern Europe, the most commonly prescribed maintenance immunosuppressive regimen for kidney transplant patients in the years 2013–2016 was triple therapy (18) comprising CNI, antiproliferative medication, and corticosteroids. The type of CNI used varied across countries, CSA ranged from 4% to 45%, and a relevant use of dual regimen (corticosteroid sparing) was shown in Slovenia and Croatia.

Other important aspects to consider in immunosuppressive therapy are the availability of several TAC formulations and the use of generics for TAC, CsA, and MMF. Specifically, TAC use is rendered complicated by a narrow therapeutic index and high pharmacokinetic variability. There are two available oral formulations of TAC: immediate release (IR) and extended release (ER). IR is formulated to promote rapid absorption and is administered twice daily, while ER is formulated with excipients that form a protective polymer coating, resulting in a slower tacrolimus dissolution rate, and it is administered once daily (19). This prolonged release has no impact on the main pharmacokinetic parameters, which remain the same for daily and twice-daily formulations (20), and several RCTs have demonstrated equivalence in terms of efficacy and safety between the two formulations (21). However, ER formulation may be particularly beneficial in improving immunosuppression compliance and subsequently long-term outcomes (22, 23).

The recent availability of generic medications on the market has allowed interchangeability with brand name products with significantly lower-cost therapies. However, generic substitution requires caution, because in transplant patients, even relatively small changes in immunosuppressive drug exposure can lead to serious clinical consequences in terms of either under- or over-exposure (24). The availability of generic formulations for CsA, MMF, and TAC, at the start of or during drug therapy, increases the complexity of treatment choice. A systematic review and meta-analysis comparing generic with branded immunosuppressive drugs in different transplant settings concluded that there was a lack of bioequivalence for the generic formulations of CsA, TAC, and MMF in transplant patients, but there was no significant difference in terms of acute rejection (25). Moreover, a recent meta-analysis reveals a lower risk of acute biopsy rejection in patients treated with generic TAC (26). In clinical practice, data on the use of different immunosuppressive formulations and generic uptake are scarce and limited to non-European countries (27).

The aim of this study is to describe the prescription pattern of maintenance immunosuppressive therapy for solid organ transplant in Italy, showing for each organ setting (the kidneys, liver, and heart) spatial and temporal variability, with a focus on generic and modified-release formulations.

## Methods

### Study design and data sources

A retrospective observational study on immunosuppressive drug utilization patterns in a cohort of transplant patients, resident in four Italian regions (Lombardy, Veneto, Lazio, and Sardinia), was performed. The cohort of the transplant patient was identified through data available in regional administrative healthcare databases linked to the national transplant information system (SIT) using a common data model and an open-source tool for distributed analyses. The study design is described in more detail elsewhere (28).

Different health information systems were used in this study:

• The hospital discharge database containing information on hospital admission and discharge, diagnoses coded with International Classification of Diseases, 9th revision, clinical modification (ICD-9-CM).
• The drug dispensing registry containing data on drugs reimbursed by the healthcare system (out-of-pocket purchases and inpatient drugs are not traced), such as the date of dispensing, number of dispensed packages, active substances, and brand names [coded with Italian market authorization code that can be associated with Anatomical Therapeutic Chemical (ATC) code].
• The inhabitant registry consisting of demographic information on patients assisted by the regional healthcare system and the dates of registration and deregistration (due to cancellation by choice, emigration or death).
• The emergency department visits database that collects data relating to emergency room visits.
• The exemptions from healthcare service co-payment database, which contains coded information about chronic diseases or socio-economic factors.

All databases can be linked at the regional level through an anonymous subject identifier. Moreover, clinical information on donor and receiving patients at different time frames are available at the national level through the SIT; to link them with the transplant cohort, an ad hoc stepwise deterministic record linkage procedure has been defined using pseudonymous information.

### Study population

The study included all patients who underwent transplant in the years 2009–2019 in the study regions, with at least one immunosuppressive dispensation post-transplant. The study enrolled only those patients who

• underwent single-organ transplant,
• were registered in the regional healthcare system,
•  survived during the 30-day post-discharge period,
• did not consume any immunosuppressive drug dispensed within 6 months prior to the date of admission for the transplant.

Transplant patients for whom linkage with SIT could not be done were excluded from the study.

We evaluated the use of post-transplant medication during the 30-day post–discharge period. The immunosuppressors that were considered were identified according to the Anatomical Therapeutic Chemical (ATC) coding classification system: CsA (L04AD01), TAC (L04AD02), MMF (L04AA06), and AZA (L04AX01) (both group antiM); EVE (L04AA18) and SIR (L04AA10) (both group mTORi); Prednisone (H02AB07), Methylprednisolone (H02AB04), and Prednisolone (H02AB06) (all three group Pred).

Based on the specific prescription patterns for the discharge period, seven regimen groups were identified: (i) CsA in monotherapy, (ii) CsA + antiM, (iii) CsA + mTORi, (iv) TAC in monotherapy, (v) TAC + antiM, (vi) TAC + mTORi, and (vii) No CNI and other combinations of immunosuppressive regimens that do not include CNI. The listed combinations can be associated with Pred dispensing (+Pred or No-Pred).

### Statistical analysis

After immunosuppressive patterns for each patient were identified, the most prescribed combination for each type of transplant was quantified.

The detected patterns were graphed by using multi-level pie charts (*sunburst graphs*), with a set of concentric rings. The size of each item proportionally denotes its contribution to the category, and this can help represent data in a hierarchical order. In our study, the first ring shows the proportion of patients treated with CsA, TAC, or without CNI (No CNI); the intermediate ring shows the proportion of patients treated in association with antiM or mTORi; the last ring shows any combinations associated eventually with Pred. TAC-based therapies are shown in blue and related gradations, and CsA-based therapies are in yellow.

In order to investigate territorial variability, the percentage of patients treated with the prevalent therapy (out of the total number of patients discharged from the facilities performing the procedure) was calculated for each discharge facility. This dimension was graphed by using box plots.

For each setting, the number of patients in the main therapeutic regimens was shown year-wise in order to analyze possible temporal variability in immunosuppressive schema.

Furthermore, an in-depth analysis was carried out on patients undergoing maintenance treatment with TAC: the share of therapies based on IR or ER drugs was identified. In addition, among MMF/CsA/TAC-based therapies, the proportion of patients treated with equivalent drugs was estimated.

## Results

We enrolled a total of 7,988 kidney, 4,488 liver, and 1,152 heart recipients. The application of the exclusion criteria to the initial selection resulted in a reduction of approximately 50% for kidney and liver transplants and more than 60% for heart transplant (Table 1). In particular, the exclusion of non-residents in the regions under study reduced the cohort size by 35% overall and similarly among different solid organ transplants; this may reflect the expertise in the field of transplantation in the study regions. It is interesting to note how the use of the exclusion criteria for subjects surviving 30 days post-discharge resulted in differences in terms of the organ transplants under study: higher mortality rates were seen for heart transplant patients than for those in the other settings. Even after the application of the exclusion criteria, as many as 4,029 kidney, 2,219 liver, and 434 heart recipients remained.

**Table 1:** Cohort enrolment: inclusion and exclusion criteria.

	Kidneys		Liver		Heart	
	*N*	%	*N*	%	*N*	%
Transplant patients (TPs) with single solid organ transplant in the study period	7,988		4,488		1,152	
TPs resident in the study region	5,318	66.6	2,942	65.6	753	65.4
TPs with incident transplant	4,596	57.5	2,675	59.6	718	62.3
TPs who survived 30 days post-discharge	4,539	56.8	2,454	54.7	599	52.0
TPs with at least one immunosuppressive prescription within 30 days post-discharge	4,335	54.3	2,324	51.8	448	38.9
TPs linked with transplant information systems	4,029	50.4	2,219	49.4	434	37.7

Some demographic and clinical characteristics differentiate incident solid transplant patients (Table 2). In heart transplant patients, the median age of recipients (50 years) and donors (39 years) is lower than in the other settings, which is due to the weight of paediatric age in this cohort; the percentage of paediatric donors (<18 years) equals 16.1% (data not provided in the table). As expected, the proportion of living donor patients reaches a non-negligible share (10%) only among kidney transplant patients. The median length (in days) of stay on admission (the kidneys: 14; liver: 19; heart: 34) also suggests heterogeneous hospitalization and different patient care types.

**Table 2:** Characteristics and combinations of prescribing patterns for patients with kidney, liver, and heart transplants.

	Kidneys (*N* = 4,029)	Liver (*N* = 2,219)	Heart (*N* = 434)
Recipient, male, *n* (%)	2,599 (64.5%)	1,717 (77.4%)	306 (70.5%)
Recipient, age, median [IQR]	54 [44–63]	56 [49–61]	50 [32–59]
Deceased donor, *n* (%)	3,608 (89.6%)	2,201 (99.2%)	434 (100.0%)
Donor, male, *n* (%)	2,157 (53.5%)	1,243 (56.0%)	276 (63.6%)
Donor, age, median [IQR]	58 [46–69]	61 [47–72]	39 [22–49]
Main indications for transplant, cause (*n*; %)			
1st	Glomerular nephropathies (1,719; 42.7%)	Cirrhosis (1,251; 56.4%)	Cardiomyopathies (257, 59.2%)
2nd	Cystic nephropathies (803; 19.9%)	Hepatocellular carcinoma (707; 32.1%)	Coronary artery diseases (110; 5.3%)
3rd	Hypertensive nephrosclerosis (347; 8.6%)	Metabolic diseases (58; 2.6%)	Congenital heart diseases (32;7.4%)
4th	Tubolar/intestinal nephropathies (273; 6.8%)	Acute hepatic necrosis (52; 2.4%)	Valvular cardiopathies (12;2.8%)
5th	Diabetic nephropathies (222; 5.5%)	Biliary atresia (46; 2.1%)	-
Remaining	Other (665; 16.5%)	Other (87; 4.0%)	Other (23; 5.3%)
Transplant hospitalization stay, median [IQR]	14 [11–21]	19 [14–28]	34 [27–51]
Charlson index, *n* (%)			
0–1	3,280 (81.4%)	762 (34.3%)	226 (52.1%)
2	612 (15.2%)	1,048 (47.2%)	136 (31.3%)
3+	137 (3.4%)	409 (18.4%)	72 (16.6%)
Main maintenance immunosuppressive therapies, combination (*n*; %)			
1st	TAC + MMF + Pred (1,674; 41.5%)	TAC + Pred (786; 35.4%)	CsA + MMF + Pred (159; 36.6%)
2nd	TAC + EVE + Pred (393; 9.8%)	TAC + MMF + Pred (591; 26.6%)	CsA + Pred (62; 14.3%)
3rd	CsA + MMF + Pred (341; 8.5%)	CsA + Pred (162; 7.3%)	TAC + MMF + Pred (40; 9.2%)
4th	TAC + MMF (300; 7.5%)	TAC (141; 6.4%)	CsA + AZA (37; 8.5%)
5th	TAC + Pred (210; 5.2%)	TAC + EVE + Pred (1,333; 6.0%)	CsA + AZA + Pred (25; 5.8%)
6th	Pred (197; 4.9%)	TAC + MMF (119; 5.4%)	CsA (22; 5.1%)
7th	TAC + EVE (125; 3.1%)	TAC + EVE (84; 3.8%)	TAC + Pred (19; 4.4%)
8th	CsA + AZA (114; 2.8%)	CsA (63; 2.8%)	Pred (15; 3.5%)
9th	CsA + MMF (87; 2.1%)	Pred (39; 1.8%)	CsA + MMF (14; 3.2%)
10th	CsA + AZA + Pred (76; 1.9%)	CsA + MMF + Pred (34; 1.5%)	CsA + EVE + Pred (10; 2.3%)
Remaining	Other (512; 12.7%)	Other (67; 3.0%)	Other (31; 7.1%)

With regard to the clinical characteristics of patients, the Charlson Comorbidity Index, a method of categorizing the comorbidities of patients based on the ICD, shows that in liver transplantation, 18.4% of patients have the highest score, in contrast to heart (16.6%) and kidney transplant patients (3.4%).

This variability is also found when considering the therapeutic combinations administered in the immediate post-discharge period (Table 2). For kidney and liver transplant patients, the most frequently administered therapeutic combination is based on TAC, the most prescribed regimen for the kidneys is triple therapy (TAC + MMF + Pred) (41.5%), and it is double therapy (TAC + Pred) (35.4%) for liver transplant recipients (in this setting, triple therapy was 26.6%). In contrast, in heart transplant patients, the role of CsA among calcineurin inhibitors is prominent, and the most prescribed combination is CsA + MMF + Pred (36.6%).

**Figure 1:**
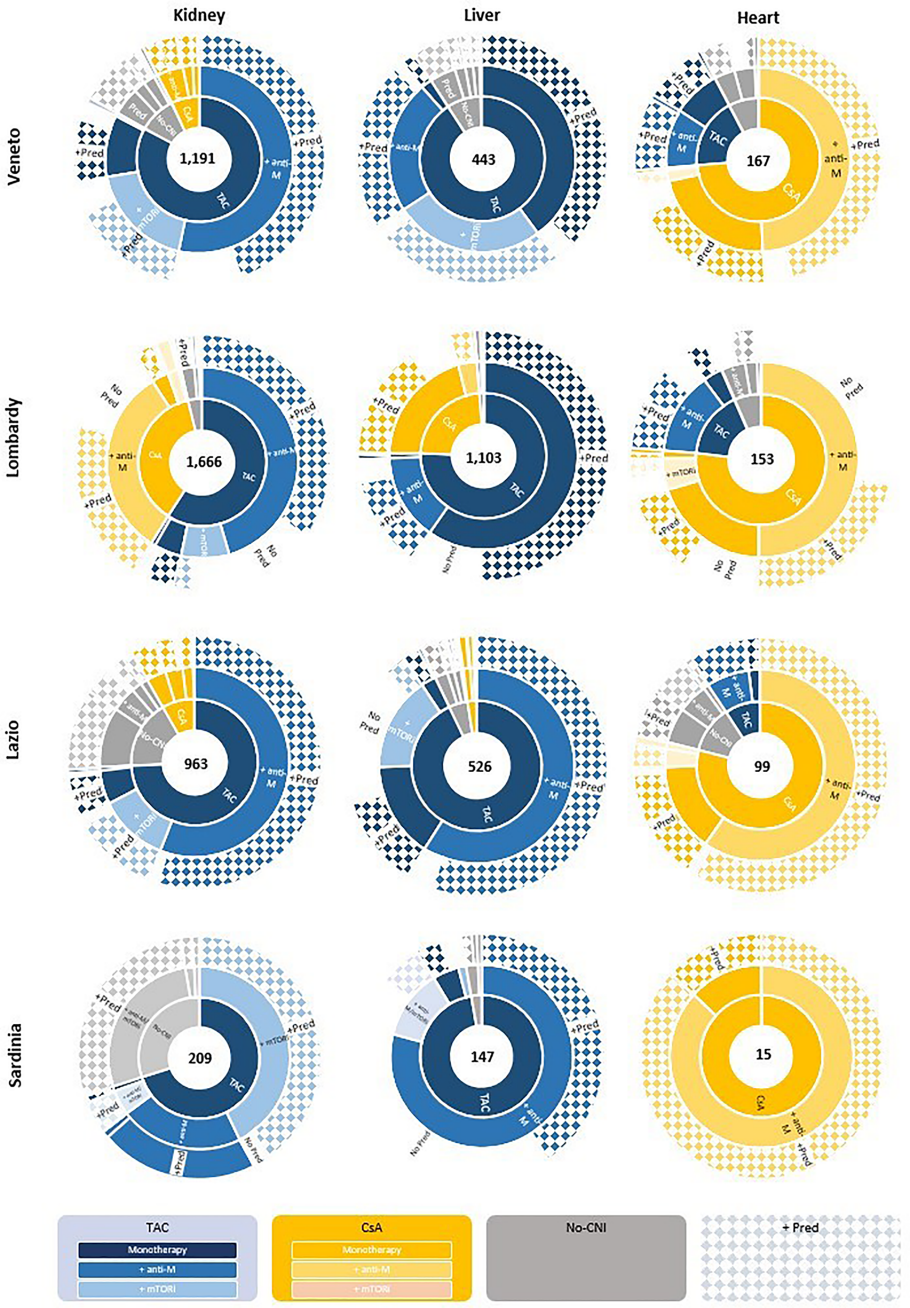
Pattern of immunosuppressive therapy by type of transplant and regions.

Through sunburst graphics and chromatic subdivision, it is possible to get a synoptic idea of the most frequently administered therapy combinations in the initial maintenance phase, taking account of transplant type and region of discharge (Figure 1). The levels of comparison can be multiple.

In the renal area, when looking at the graphs vertically, it is interesting to note that there is regional variability in patients treated with CsA (Sardinia 0%; Veneto 7.3%; Lazio 8.2%; Lombardy 37.3%). This difference also emerges with regard to liver transplantation (Sardinia 0%; Veneto 0.4%; Lazio 2.3%; Lombardy 23.5%). While the heart setting shows a similar pattern in the use of CNI-based therapy for all regions in the examination of the graphs, CsA is the most commonly administered medication in heart transplant patients.

Furthermore, variability, both by organ setting and by region, can be noted in the drugs used in combination with CNIs. In particular, in the kidney setting among TAC-based users, a significantly larger proportion of patients are treated with mTORis in Veneto and Sardinia compared with Lombardy and Lazio; while for liver recipients, the use of mTORis is higher in Veneto than in the other regions.

In the context of the heart, CsA-based therapies, either alone or in combination with antiM and mTORi, are globally matched by Pred use, except in Lombardy, where a higher proportion of patients are treated without Pred (36.8%).

**Figure 2:**
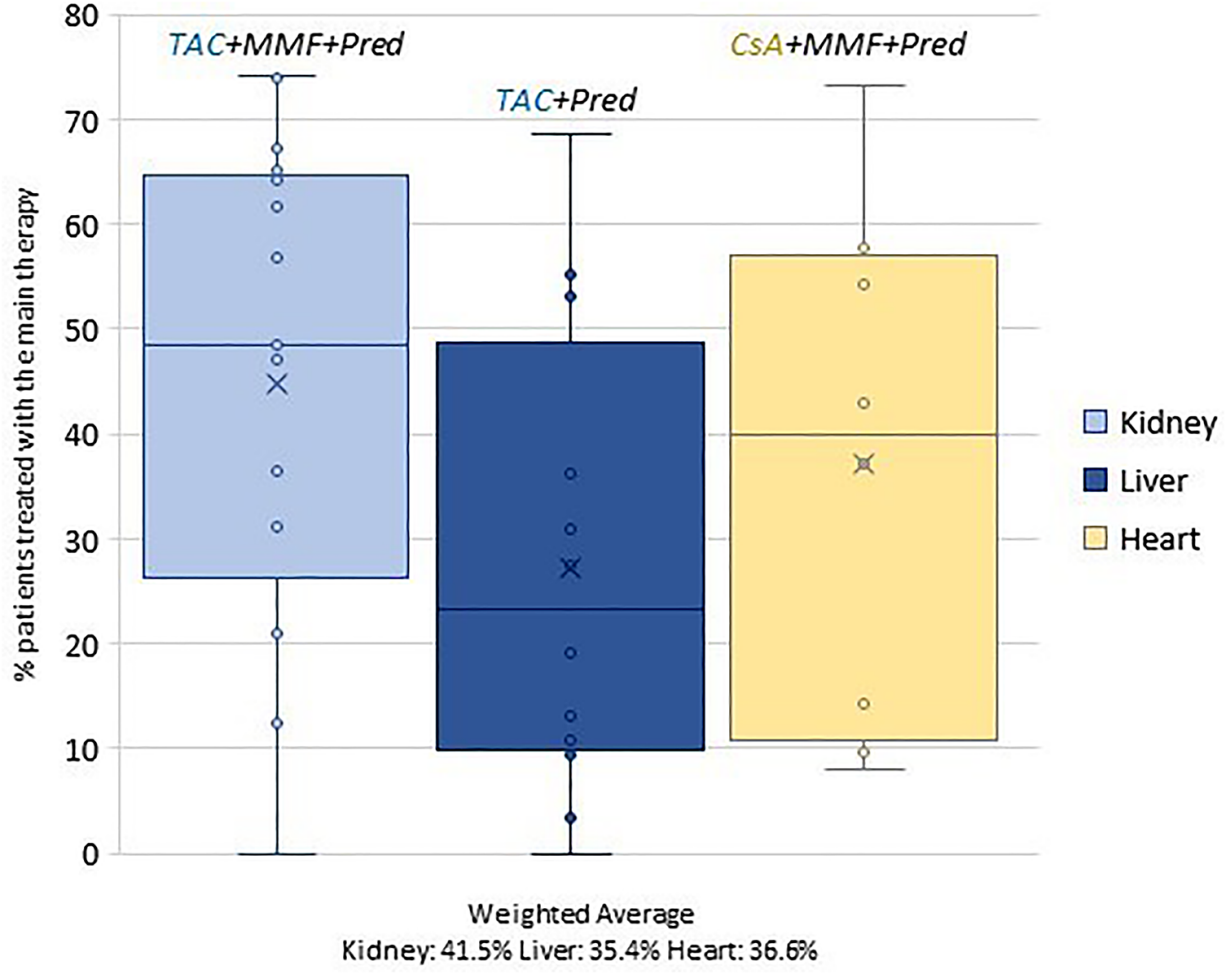
Variability by discharge facility of the main drug therapy by organ.

Differences observed by region reflect an important heterogeneity that is linked to centres (Figure 2). In the case of the kidney cohort, for example, the percentage of patients treated with triple therapy (TAC + MMF + Pred) ranges from 0% to 74%.

**Figure 3:**
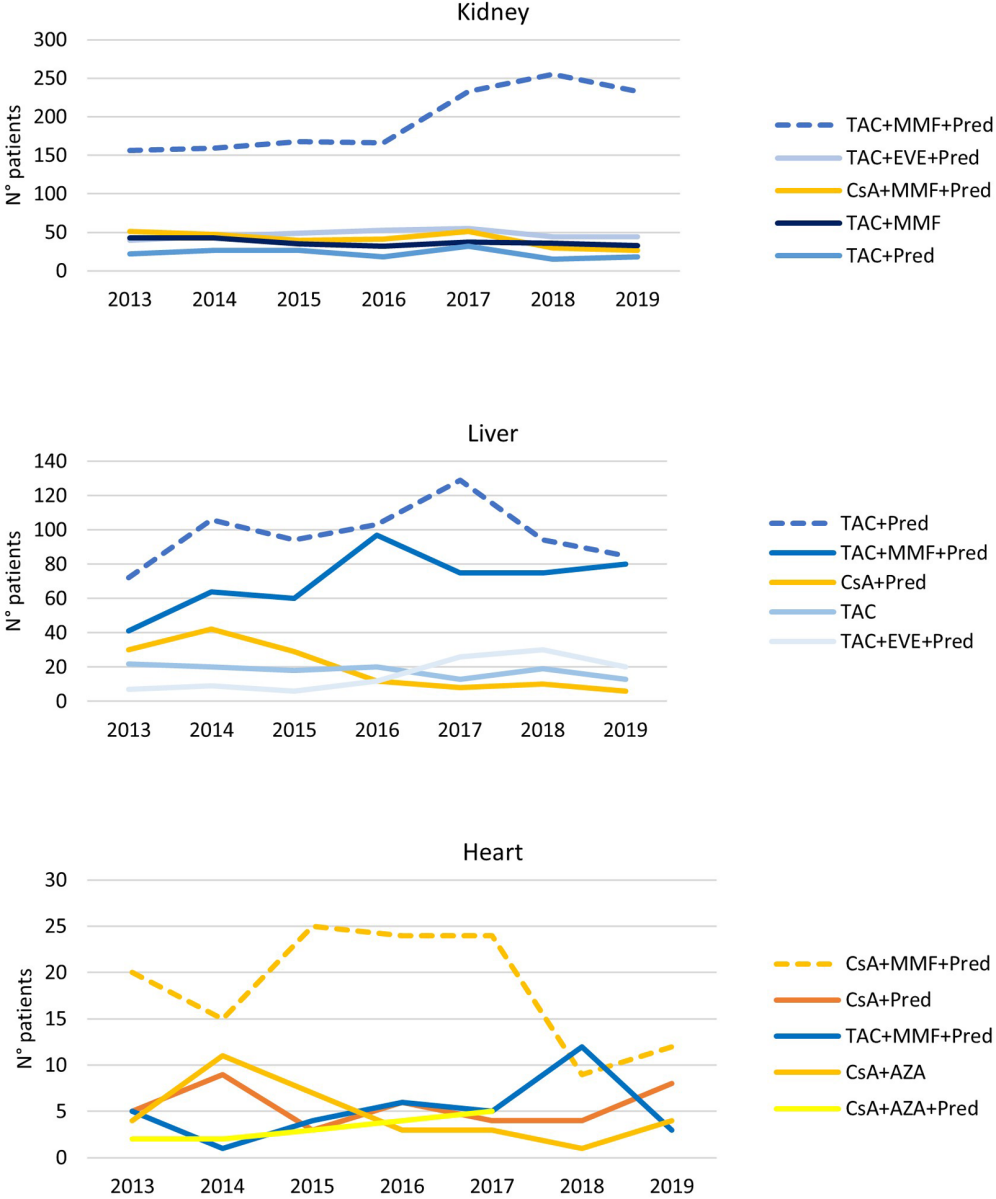
Immunosuppressive therapeutic combination over the years in kidney, liver, and heart transplantations. The analysis has been limited since 2013, when data for all regions were available.

The trend analysis, presented in Figure 3, shows that, for kidney and liver transplants, there is a progressive increase in immunosuppressive TAC-based therapies (the kidneys, 2013: 63.0%, 2019: 82.2%; liver, 2013: 72.7%, 2019: 92.9%) at the expense of CsA-based therapies. In particular, in the case of liver transplantation, the most frequent index therapy is the combination of TAC and Pred that remained stable over time, while triple therapy shows an increase from 18.1% in 2013 to 31.3% in 2019. In the case of heart transplantation, the most frequently administered therapy remained triple CsA-based therapy (CsA + MMF + Pred) during the whole period of observation. In recent years, there has been a slight decrease in the percentages of these therapy protocols (2013: 43.5%, 2019: 29.3%) at the expense of a slight increase in TAC-based therapies.

The use of generics and extended-release tacrolimus by organ type and region is shown in Table 3. A comparison of patients treated with TAC and MMF revealed a higher proportion of patients treated with generic drugs in Lombardy than in the other regions, regardless of the type of transplant done (Table 3). The generic version of CsA is mainly used in Veneto for heart recipients, while an underuse of generic TAC is reported in Sardinia for renal transplant patients.

**Table 3:** The use of generics and extended-release tacrolimus by organ type and region.

	Kidney							Liver							Heart						
	TAC			CsA		MMF		TAC			CsA		MMF		TAC			CsA		MMF	
	*N*	%Gen^a^	% ER^b^	*N*	%Gen^a^	*N*	%Gen	*N*	%Gen^a^	% ER^b^	*N*	%Gen^a^	*N*	%Gen^a^	*N*	%Gen^a^	% ER^b^	*N*	%Gen^a^	*N*	%Gen^a^
Veneto	991	10.0	65.5	87	-	748	25.5	402	26.6	53.2	2	-	121	40.5	31	12.9	-	123	35.0	90	26.7
Lombardy	984	65.5	19.2	621	48.0	1,141	68.7	833	45.4	20.6	257	18.3	201	89.6	26	26.9	-	117	13.7	72	94.4
Lazio	714	12.2	58.1	79	2.5	606	5.8	489	17.2	40.1	12	-	334	33.5	9	22.2	11.1	78	1.3	64	17.2
Sardinia	146	0.7	100.0	-	-	113	36.3	143	12.6	-			137	8.8				15	6.7	12	33.3
Total	2,835	29.3	49.3	787	38.1	2,608	40.3	1,867	31.4	31.2	271	17.3	793	44.5	66	19.7	1.5	333	18.3	238	45.0

^a^ Patients treated with the generics of tacrolimus.

^b^ Patients treated with extended-release tacrolimus.

The use of extended-release TAC-based therapies varies widely between both transplant types and regions. For both kidney and liver transplantations, Lombardy has significantly lower extended-release treatment rates (kidney: 19.2%, liver: 20.6%) compared with Lazio and Veneto.

## Discussion

To the best of our knowledge, this is the largest European cohort study on immunosuppressive drug therapy in the setting of transplant patients (more than 6,600 transplants were carried out in the 2009–2019 period). As expected, demographic characteristics differed between patients with kidney, liver, and heart transplantations. The number of female recipients was higher in kidney transplants, and patients with heart transplants were of younger age. A higher comorbidity score was found for kidney recipients, while the highest Charlson index was reported for liver recipients. Furthermore, a high spatial/temporal variability in prescription patterns for immunosuppressive drugs in relation to organ setting and region of assistance emerged.

As shown by our results, in kidney transplants, the most prescribed therapy is the combination of TAC + MMF + Pred with an important variability across regions, potentially attributable to specific therapy protocols implemented in centres. In particular, a higher use of CsA-based therapy was detected in Lombardy, while in Lazio and Sardinia, a considerable proportion of patients were treated with a regimen without CNI. These clinical practice immunosuppressive regimens seem to be at variance with published evidence showing the net benefits of TAC (10) and suggesting the use of triple therapy (TAC + MMF + Pred) (4, 18, 29). It is important to underline here that the proportion of patients treated with triple therapy has grown over time, and this could indicate an evolution of clinical protocols, tied to evidence, during the time span considered.

In liver transplants, the most frequent immunosuppressive regimen is double therapy (TAC + Pred), followed by triple therapy (TAC + antiM + Pred). This ranking is reversed in Lazio and Sardinia, and the use of the combination TAC + mTORi + Pred is frequent in Veneto.

A great heterogeneity in the immunosuppression protocols used in different transplant centres was described in a recent article, which provided recommendations on the therapeutic schema to be adopted in this context. This took into account the type of liver recipient: standard patient, critical patient, patient with a specific aetiology, patient with hepatocellular carcinoma, patient with de novo malignancies, etc. In many cases, it is possible to adopt more than one combined immunosuppressive strategy, as no differences in the risk–benefit profile have been demonstrated (30).

However, our clinical practice data showed an increase in the use of triple therapy (TAC + MMF + Pred) over time in the liver setting, with the patient figures reaching a similar number treated with double therapy (TAC + Pred) in 2019, while a decrease in CsA-based therapies was observed from 2016 onwards.

In heart transplant, the most prescribed immunosuppressive regimen was CsA-based, with 36.6% in triple therapy (CsA + MMF + Pred) and 14.3% in double therapy (CsA + Pred). Regional differences showed a lower proportion of patients treated in combination with Pred in Lombardy and a higher use of TAC-based therapies in Veneto and Lombardy, particularly in the last study years. For this kind of transplant, the available evidence on the optimal immunosuppressive strategy is less robust, even though the more recent recommendations have suggested the TAC-based regimen (31).

Spatial variability concerns not only the number and type of drugs used but also the use of generics and the formulations dispensed. Specifically, Lombardy shows the highest use of the generic version of TAC for all organ settings, while the Veneto region shows that TAC ER is frequently dispensed for kidney and liver transplants. Such differences in immunosuppressive treatments across the country could have repercussions on both national healthcare sustainability and patient compliance.

### Strengths and limitations

The main strength of our study is the availability of immunosuppressive dispensations in four different Italian regions, which are representative of all geographical areas (Veneto and Lombardy for northern Italy, Lazio for the centre, and Sardinia for the south) and where more than 45% of transplant activity take place. To our knowledge, this is the largest and most representative population-based study illustrating the maintenance immunosuppressive regimen post-transplant in Italy. A limitation of the study is related to the administrative nature of our data: only prescriptions of medicines reimbursed by the Italian National Health Service were detected; however, it is highly probable that the use of non-reimbursed medications in the transplant population is limited.

## Conclusion

The CESIT study is the first Italian multicentre study that focuses on immunosuppressive treatment post-solid organ transplantation and has documented high spatial and temporal variability in the immunosuppressive protocols adopted. Monitoring the immunosuppressive strategy for each organ by region and year is the first step to providing valuable information on the actual management of drug therapies in clinical practice. Further studies are needed to investigate the determinants of this variability in terms of patient and centre characteristics and to compare whether different drug therapy schemes have the same benefit–risk profile in clinical practice.

## Data Availability

The datasets presented in this article are not readily available because the datasets generated and/or analyzed during the current study are not publicly available because of privacy reasons. Requests to access the datasets should be directed to v.belleudi@deplazio.it.

## CESIT working group

Antonio Addis, Nera Agabiti, Valeria Belleudi, Massimo Cardillo, Paolo Carta, Marina Davoli, Michele Ercolanoni, Eliana Ferroni, Pamela Fiaschetti, Marco Finocchietti, Bedeschi Gaia, Donatella Garau, Valentina Ientile, Ursula Kirchmayer, Luca L’Abbate, Stefano Ledda, Lorella Lombardozzi, Olivia Leoni, Ersilia Lucenteforte, Claudia Marino, Maria Lucia Marino, Lucia Masiero, Marco Massari, Arianna Mazzone, Ugo Moretti, Arianna Bellini, Maurizio Nordio, Alessandra Oliveti, Daniela Peritore, Giuseppe Piccolo, Silvia Pierobon, Francesca R. Poggi, Francesca Puoti, Andrea Ricci, Alessandro C. Rosa, Vito Sparacino, Stefania Spila Alegiani, Silvia Trapani, Gianluca Trifirò, Martina Zanforlini, Manuel Zorzi.
